# “*With all your friends in your pocket*” – a qualitative study about adolescents’ experiences with social media from a health-promoting perspective

**DOI:** 10.1080/17482631.2026.2639536

**Published:** 2026-03-06

**Authors:** Trude Vie Ytrearne, Randi Træland Hella, Gunnhild Johnsen Hjetland, Jens Christoffer Skogen, Eva Langeland

**Affiliations:** a Department of Health and Caring Sciences, Faculty of Health and Social Sciences, Western Norway University of Applied Sciences, Bergen, Norway; b Regional Centre for Child and Youth Mental Health and Child Welfare (RKBU Vest), NORCE, Bergen, Norway; c Department of Health Promotion, Norwegian Institute of Public Health, Bergen, Norway; d Centre for Evaluation of Public Health Measures, Norwegian Institute of Public Health, Oslo, Norway; e Center for Alcohol and Drug Research (KORFOR), Stavanger University Hospital, Stavanger, Norway

**Keywords:** Adolescents, social media, health promotion, qualitative research, youth mental health

## Abstract

**Purpose:**

To explore adolescents’ experiences with social media (SoMe) use from a salutogenic health-promoting perspective.

**Methods:**

This qualitative study was based on five focus group interviews (27 adolescents) from two public senior high schools in Norway, (15–18 years). Interviews were analyzed using thematic analysis.

**Results:**

SoMe-use was described as an important part of the adolescents` everyday life and a novel way to relate to the world. The identified main themes were: social support, self-esteem, and their interplay. They experienced social support via SoMe through communication, feeling unity, inclusion, and the establishment of a larger social network, thus preventing loneliness and promoting social belonging. Further, SoMe-use is important for their self-esteem by receiving and providing attention, acceptance, and confirmation. Social support promoted their self-esteem, which in turn enhanced their online self-expression, further reinforcing the social support.

**Conclusions:**

These findings indicate that SoMe might be an important social arena for the development of adolescents’ self-esteem and receiving social support. Further, it seems that there is an interplay between social support and self-esteem that might positively influence their identity, sense of coherence, and mental health. However, we need more in-depth knowledge about adolescents’ experiences with SoMe-use from a salutogenic health-promoting perspective.

## Introduction

During the last decade, social media has become ubiquitous in the lives of adolescents (Webster et al., 2020). Social media plays a key role in adolescents’ everyday lives (Bakken, 2020) and has, for many, become a significant aspect of their lived experience, though its impact varies across individuals depending on access, skills, and personal context (Saboga-Nunes et al., 2022). Social media enables adolescents to be in touch and socialise with others without being physically close (Bakken, 2020). Social media constitute central communication platforms for what happens in youth culture, for schoolwork, and for adolescents’ social life in general (Hagen & Wold, 2017; Nyjordet, 2018) and has increasingly become a central feature in the lives of adolescents (Nesi et al., 2020). Social media can facilitate relationships between people from different backgrounds, which can result in richer social interactions (Kapoor et al., 2018). Through social media, adolescents keep in touch with existing friends and establish new friendships (von Tetzchner, 2012), increasing their size and diversifying the composition of their social networks (Best et al., 2014).

In the present study, the following definition of social media was used: “Social media employ mobile and web-based technologies to create interactive platforms via which individuals and communities share, co-create, discuss and modify user-generated content” (Kietzmann et al., 2011, p. 1). Adolescents are spending an increasing amount of time online (Odgers & Jensen, 2020). The Norwegian Media Authority (2024) found that 86% of Norwegian 9–18-year-olds are on one or more social media platforms, and Bakken (2024) reported that over one-third spend three or more hours on social media every day, with girls spending more time on social media than boys. Overall, young people are still the most active and frequent users of social media (Pew Research Centre, 2021; von Tetzchner, 2012).

Despite the rapid growth in social media use among adolescents, in-depth knowledge of how they experience participation in social media is still lacking. A systematic review of all studies that investigated the relationship between subjective well-being and social networks (both online and offline) revealed both positive and negative association between social networks and the subjective well-being of adolescents (Webster et al., 2020). The study revealed that online social networks had a positive association with mood, life satisfaction, and loneliness through support-seeking behaviour and receiving positive feedback. Despite the potential positive effects of social media use on well-being, most published studies focus on the potential negative effects of social media use, and very few studies are able to identify the potential positive effects of social media use if they exist (Schønning et al., 2020).

Social media use related to adolescents’ mental health from a health-promoting perspective is a new field of research with a dearth of knowledge. In Norway, an important white paper focusing on child and adolescent mental health emphasised that we need more knowledge about how social media use influences mental health among adolescents (Ministry of Health and Care Services, 2019). A national strategy has been developed to consider the positive aspects and opportunities of internet use by children and adolescents, as well as risks and challenges (Ministry of Children and Families, 2021).

In summary, there are various nuances and knowledge gaps concerning adolescents’ social media use and their mental health. Over the past decade, attention has focused on the negative effects of social media on mental health, while positive effects have received less emphasis (Schønning et al., 2020). Quennerstedt (2020) argued that social media can also serve as a health-promoting resource for adolescents, supporting the need for research exploring salutogenic experiences of social media use. Therefore, more research is needed, especially concerning health-promoting experiences on social media. The main aim of the present study was to explore the experiences of social media use among adolescents from a salutogenic health-promoting perspective.

## Theoretical framework

Health promotion has been defined by the World Health Organisation (WHO) as “the process of enabling people to increase control over and to improve their health” (World Health Organization, 1986). This definition is compatible with the theory of salutogenesis, which focuses on the factors that support good human health, rather than the origin of disease. In the present study, the basic principles and concepts from the theory of salutogenesis are applied to understand social media from a health-promoting perspective (Antonovsky, 1987, 1996).

In salutogenesis, all persons are seen and understood as having different levels of health along a continuum. The main question is what explains the movement of the health ease/disease continuum. One answer is generalised resistance resources (GRRs), which may be referred to as “a property of a person, a collective or a situation which, as evidence or logic has indicated, facilitated successful coping with the inherent stressors of human existence” (Antonovsky, 1996, p. 15). Furthermore, “GRRs foster repeated life experience which help one to see the world as ‘making sense’, cognitively, emotionally and instrumentally” (Antonovsky, 1996, p. 15). An overall GRR is a sense of coherence (SOC), which plays a central role in movement along the health continuum. SOC is an orientation that contributes to viewing life activities and events as comprehensible, manageable, and meaningful (Antonovsky, 1987, 1996). The strength of one’s SOC is promoted by one’s ability to use different GRRs, which contribute to life experiences that include consistency, underload‒overload balance and participation in decision-making (Antonovsky, 1996). This means that the higher degree of personal and collective GRRs, the individual has available and can use, the stronger the SOC and the higher the degree of health, especially mental health (Langeland & Vinje, 2022). However, while SOC may develop throughout the entire life course, it is especially sensitive to changes during adolescence (Braun-Lewensohn et al., 2022). Importantly, social media can be an important resource in a salutogenic perspective by providing adolescents with access to comprehensible information, social support, and tools for coping, as well as meaningful social connections. This can enhance their coping, abilities, SOC, and mental health.

In a digital lifeworld, social media can be compared to a library that is continually receiving new “books” (Saboga-Nunes et al., 2022) and thus is a major GRR because it provides adolescents with access to resources on how to cope with life challenges, builds a strong social base, and can promote psychological well-being in the digital lifeworld.

Two of the most important direct GRRs for developing SOC are the quality of social support and identity. People who have close social relationships solve tensions more easily than those who do not have these close relationships (Langeland & Vinje, 2022). Weiss (1974) shared Antonovsky’s view of the importance of the quality of social support for health. He identified six social needs that might be fulfilled by relating to others: attachments, social integration, opportunity to provide nurturance, confirmation of self-worth, reliable alliance, and guidance from others. During adolescence, social support is particularly crucial, as it provides both emotional safety and a context for the development of identity. In addition, cognitive resources such as knowledge and reflective awareness enable adolescents to make informed choices and interpret their actions meaningfully. This process enhances the experience of coping and reinforces both identity and a sense of coherence (Langeland & Vinje, 2022).

## Methods

### Research design

The present study is part of a larger innovation project in Bergen Municipality. The project explores the links between activity on social media and adolescents’ mental health and well-being (Folkehelseinstituttet, 2022; Hjetland et al., 2021; Ung på sosiale medier (u.å)). The present study used a qualitative design, including five focus groups (FGs) (Malterud, 2018; Polit & Beck, 2017), to explore adolescents’ experiences of social media use from a health-promoting perspective. The empirical material used in this study has also been used in previously published articles; however, it has different analytical purposes. These earlier studies have primarily focused on associations between adolescents’ use of social media and their mental health and well-being. The present study adopts a health-promoting qualitative perspective. This entails an analytical shift toward resources, strengths, and social media as supportive processes in adolescents’ everyday lives, with particular attention given to coping, social support, identity development, and experiences of belonging. Thematic analysis was used to analyse the data (Braun & Clarke, 2006), and more details are given below.

### Sample

Participants were recruited from two public senior high schools in Bergen, Norway. Five focus groups were recruited from the two schools (Hjetland et al., 2021). To ensure the validity of the study, strategic selection was performed by recruiting from one school located in the city centre and one located in a rural area with a different socio-demographic profile. General studies and different vocational education training programmes were offered at both schools. One of the schools had an e-sports programme. Teachers at each school were informed about the study and how to participate. Adolescents who wanted to participate and were motivated to contribute with reflections and discussions about social media were then recruited. Twenty-seven adolescents from 15 to 18 years old (mean age, 16.8 years) participated, and each group included 5–6 participants. None of the participants withdrew from the study during or after the interview session. Gender is regarded as a dimension that can influence group dynamics and is a key prerequisite for optimal homogeneity. Therefore, single-gender focus groups were predominantly used to reduce potential social discomfort in mixed settings and to encourage more open discussion. As a result, two focus groups consisted of female students only and two focus groups of male students only, while one focus group included both male and female students. However, the use of single-gender focus groups was intended to facilitate group interactions rather than to introduce gender as an analytic unit in the study. Anonymized transcribed data from the project were used in this study.

### Data collection

The interview guide was developed based on an empirically and theoretically informed framework, drawing on themes identified in an ongoing scoping review on social media, mental health, and adolescent well-being (Schønning et al., 2019, 2020), methodological guidance for qualitative interviewing (Malterud, 2018), and input from a resource group. A previously developed interview guide (Schønning, 2018) served as a template and was adapted to the study context. Development involved the project group, a resource group of seven older adolescents (16–18 years) from the municipal Youth Council, and four master-level psychology students under supervision. The interview guide focused on adolescents’ use of social media and their perceptions of it as both a positive and negative influence in their lives. The resource group provided feedback and piloted the guide to ensure relevance and clarity from a youth perspective, while the psychology students supported the literature review, adaptation, draughting, piloting, and revisions to ensure methodological quality. The completed guide was piloted on the resource group, which led to some additional changes, such as question wording, to the final interview guide (Hjetland et al., 2021).

Five focus group interviews were conducted in 2019. The focus group interviews consisted of two 40-minute sessions with a break in between. The focus group interviews were audiotaped and then transcribed verbatim. The project leader (RTH) and one researcher (GJH) facilitated the focus group interviews, acting alternately as moderator and secretary for each focus group interviews. The moderator presented the topic, facilitated the discussion, and tried to create a benevolent and open atmosphere. To ease the transcription of the focus group interviews, the secretary wrote down the first few words of each sentence and who said it. They also noted aspects of the focus group interviews that were difficult to capture on tape, such as non-verbal communication and the social climate of the group.

### Analysis

In this study, the interviews were thematically analysed (Braun & Clarke, 2006) to identify themes or patterns in the collected data. Reflexive thematic analysis is a useful, flexible method for qualitative research with six phases. The analyst moves through these phases in an iterative manner, continuously revisiting and reflecting on earlier phases as the analysis progresses (Braun & Clarke, 2019): The material was manually coded. Different colours were used to highlight text segments corresponding to emerging themes and patterns, allowing for the identification and organisation of key concepts across the dataset.

**Phase 1.** One researcher (TVY) read and re-read the material to become familiar with the depth and breadth of the content. EL and RTH also read the material to obtain an impression of the content. Potential topics and the drafting of ideas were discussed in meetings with RTH, EL, and TVY, and potential themes were noted.

**Phase 2.** During the analysis, one researcher (TVY) identified text related to the salutogenic health-promoting perspective relevant to this study. The identified text was discussed among EL, RTH, and TVY and was marked in thematically organised colour codes, which further structured the interview data. There was also a meeting with the whole research group where findings were discussed and confirmed.

**Phase 3.** TVY sorted the coded material into potential themes where findings were inserted into spreadsheets, and TVY began to look for repeated meaningful sub-themes and patterns. The findings were discussed in a meeting with EL and RTH.

**Phase 4.** To review the themes against the sorted coded text segments in Phase 3, TVY plotted them into a diagram. It then became clearer which statements of opinions (i.e., sub-themes) gave few answers (i.e., narrow) and which statements of opinion gave many answers (i.e., broad). The findings were discussed in a meeting with EL and RTH.

**Phase 5.** A satisfactory theme map of the data was reached. Through the analysis, the researchers EL, RTH, and TVY formed an impression of the themes that could promote answers compatible with the purpose of the study. The themes were defined and given final names in collaboration with the entire research group.

### Ethical considerations

All participants gave their informed consent. The participants were informed that they could withdraw at any time and could choose how active they wanted to be in the discussions. The interviewers took care to address their experiences with respect and dignity. There was no indication that the adolescents in the FGs felt pressure to participate. However, adolescents were recruited in a school context, and the teacher‒pupil relationship or general psychosocial classroom environment may have influenced their willingness to participate. All participants’ names were replaced with pseudonyms when the interviews were transcribed. Other identifiable information, such as the school they belonged to, and their usernames on social media, was omitted or replaced. Transcribed interviews were only available for those directly involved in the transcription, coding, and analysis of the material. Audio files were permanently deleted following transcription. Data were stored on the Bergen Municipality’s secure storage and archive system.

### Ethics approval and informed consent to participate

All procedures in this study were conducted in accordance with the ethical standards of the institutional and national research committees, and with the principles outlined in the Declaration of Helsinki (WMA Declaration of Helsinki).

As the study fell outside the remit of the Norwegian Act on Medical and Health Research, approval from the Regional Committees for Medical and Health Research Ethics (REK) was not required. Instead, approval was obtained from the Data Protection Officer at the Section for Digitalisation and Innovation, Department of Finance, Business Development and Property Management, Bergen Municipality, in accordance with GDPR regulations (reference 2019/66930). The project also included risk and vulnerability analysis, including ethical considerations, led by the project manager in Bergen Municipality. An ethical assessment was also performed by the project group.

According to Norwegian regulations, no further research ethics approval was necessary, as this project did not involve medical interventions, the collection of biological material, or the processing of sensitive personal health data.

In line with the guidelines of the Sikt – Norwegian Agency for Shared Services in Education and Research (formerly NDS), individuals aged 15 and above may provide informed consent to participate in research that does not involve bodily intervention or the processing of sensitive personal health data. This is consistent with national ethnical guidance from the Norwegian National Research Ethics Committees, which recognises adolescents aged 15 and older as capable of providing informed consent in certain types of social science and health-related research.

All participants in this study provided written informed consent prior to participation. They received both oral and written information about the study’s purpose, procedures, data handling, and rights, including the right to withdraw at any time, either verbally or in writing. The consent process included clear communication about the audio recording of interviews, which was explained during the initial meeting, reiterated orally at the time of the interview, and documented in the written consent form.

## Results

**Figure 1.**
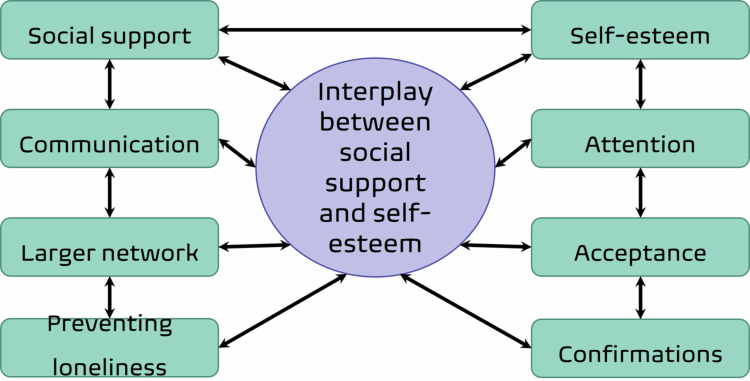
A health-promoting perspective illustrating the dynamic interplay on social media between the overall theme, main themes, and associated sub-themes.

All participants reported that social media was an important part of their lives. They compared it to being interconnected in a world parallel to the physical world. The participants’ experiences converged under the overall theme, “the reciprocal interaction between social support and self-esteem”, along with two main themes, “social support” and “self-esteem” (Figure 1).

Within each of the main themes, several sub-themes were developed and named as the participants expressed them or were gathered under a common concept. The conceptual model thus illustrates the reciprocal relationship between social support and self-esteem, emphasising key contributing factors within each domain. The arrows indicate the bidirectional nature of these interactions.

### Interplay between social support and self-esteem

As presented in Figure 1, the analysis revealed an interplay between social support and self-esteem that seems to be health promoting. For example, being part of a chat group (Snapchat) gave adolescents the opportunity to communicate; receiving feedback and feeling that they were important to others made them feel good. The following quote highlights this interaction: *“Your friends support you, and that makes you kind of feel better*” (FG1). The participants discussed how many adolescents struggle with self-esteem during adolescence and reported that experiencing support from friends on social media could promote self-esteem. In turn, better self-esteem encouraged them to be more open and express themselves more distinctly.

### Social support

The participants discussed several ways in which social media could provide the experience of social support. Four sub-themes were generated: (1) communication, (2) unity and inclusion, (3) larger networks, and (4) preventing loneliness.

## Communication

All the study participants expressed that they used social media as a communication platform that offered new ways to maintain contact with others regardless of physical distance and time of day. The participants reported using social media as a global, different, and easier way to communicate than the traditional forms of communication outside of social media: “*It is easier to communicate with people. I have friends all over the world. Using social media makes it easier to stay in touch with my friends and talk to them*” (FG4).

Further it seems that there is a seamless integration of conversations in social media and in real life: “*Many of the conversations I have with my friends in reality are based on the conversations on social media*” (FG1), or as another participant expressed: “*you continue the same conversation there*” (FG1).

## Unity and inclusion

The participants expressed that being part of a social group or network could foster a sense of belonging and inclusion, making it easier to experience unity and connection compared with other areas of their lives. One participant said: “*By using social media I feel you get better unity with more people, since social media makes it easier to keep in touch with several people at the same time*” (FG4). Some participants also described social media use as a function and resource they needed to maintain contact with each other: “*a resource everyone needs, in a way, to reach each other*” (FG4). Social media provides an opportunity to maintain contact with friends and family, regardless of physical encounters. In addition, social media provided participants with a sense of control over what was happening around them: “*Feeling of being in control … of what is happening around you*” (FG5), or as another participant expressed: “*it gives you better mental health and, because you do not stress, you have control over your mind, in a way*” (FG5). This sense of control enables participants to engage actively in social networks, maintain contact, and maintain friendships across the world. In turn, this strengthened their feelings of unity and inclusion, as active participation and self-directed engagement in social relationships promoted a feeling of belonging and social support. It is important to distinguish between friendships that exist in “real life” and are also present on social media, and friendships that exist solely online. The two types of relationships are qualitatively different: real-life friendships often involve greater emotional closeness and trust, whereas online-only friendships provide social connections in a more situational or fragmented way.

## Larger social networks

Adolescents experienced that social media enabled them to create larger social networks than they did outside of social media. The participants described social media as a social arena with other norms and rules; a different culture compared to the other social arenas they were part of, mostly due to the written communication forms usually employed. It was easier to connect, develop new friendships and make acquaintances on social media than in real life, mainly because of access to a wider community all over the world. The participants described “others” as adolescents who had the same interests as themselves or someone they could discuss with: *“I have, in a way, got to know new people. They may disagree or agree with your opinions, but you can get someone to talk to and in that way, you get a larger network”* (FG1). In general, participants described social media use as a positive resource that helped them increase the scope of social circles that could promote the experience of social support.

## Preventing loneliness

Our findings showed that participants’ experiences of always having other adolescents available online via social media were positive resource that prevented loneliness: “*Many people feel lonely, and then social media can be positive for you, if you find the right platforms*” (FG2). Further, they described social media as a resource where they always had someone to talk to: “*In a way you have the opportunity to talk to your best friends all the time, talk to others that can help if you need it*” (FG2), or as one girl said: “*You always have your friends in your pocket*” (FG1). Some participants described social media as a resource they used when life was difficult, allowing them to reach out more safely, receiving social support, and maintaining connection and belonging even without direct interactions. One participant stated that it allowed them to “*talk to people you love, when you need it*” (FG4). They also described using social media (YouTube, TikTok) to prevent negative thoughts, which they believed would provide a positive health-promoting benefit: “*When I watch YouTube or other entertainment on social media, it takes away the focus on what made me in a bad mood*” (FG3). This use of social media can also be understood as a strategy to prevent feelings of loneliness, as engaging with content or participating in online communities provides a sense of connections, distraction from negative emotions, and indirect social engagement, thereby supporting psychological well-being. Overall, the participants expressed that feedback and conversations with “others” gave them a feeling of social support and prevented loneliness. Further, they encouraged informing parents, schools, and others that the use of social media does not necessarily lead to poor mental health but can play a positive role in adolescents’ lives because of increased possibilities for socialising, thus preventing loneliness.

### Self-esteem

During the interviews, the participants discussed how social media affected their self-esteem. Three sub-themes were developed: (1) attention, (2) acceptance, and (3) confirmations.

## Attention

The participants said that social media was a source of attention from others. This was partly ascribed to the different set of norms or rules of communication on social media, lowering the threshold for contacting others, and thus increasing opportunities to gain attention from peers: “*It is the same as, I could not go to school and sort of say hello to a person just because, as I said before, there are other rules there. So it is in a way, you kind of have several ways to get attention”* (FG1). The participants expressed that attention, feedback, and good conversations with several available others who listened to them, gave them a good feeling, which in turn affected their self-esteem: “*social media can, in a way, give me a good feeling in the form that I get better self‐esteem. Because I notice that … you usually have good conversations with people there and you get attention, in a way, all the time. Because you have so many people all the time”* (FG1).

## Acceptance

In this study, we refer to acceptance as being valued and included for the person one is, which can make it easier to express oneself and experience social belonging. Many social media platforms are partly based on written communication (Snapchat, Discord, Instagram, Messenger). Videos and pictures are also common. Several of the participants discussed how it was easier to express themselves and their opinions in written communication than in face-to-face interactions outside of social media, where they were unable to express themselves as they wished. Further, some of the participants expressed that the use of social media provided a better impression of other people and that it was easier to be heard and accepted on social media regardless of extroverted or introverted personalities: “*They get to know your personality in another way*” (FG3). Another participant expressed that it was “*a different way for people who tend to be a bit shy to be more social*” (FG3). Generally, the participants described that being accepted for who they were on social media could help promote their self-esteem, which again encouraged them to be more open and express themselves more distinctly.

## Confirmation

With confirmation, we refer to explicit feedback from others, which validates one’s actions or opinions and can strengthen self-esteem and the experience of social belonging. The participants observed that social media (Instagram, TikTok, Snapchat, story replies, emoji reactions, and tagging) could help promote self-esteem during adolescence through confirmation from others:

“*Yes, because at our age, what most people struggle with, is often related to their self‐esteem. And that is why, when you are sad and such, then you seek confirmation, right, and then you hear:* ‘*No, but you are so incredibly nice*’”. (FG1)

Participants also said that getting confirmation from several others on social media, gave them the experience of feeling they were important to others, which made them feel good: “*then you feel like, that person has actually clicked on my name for me to be part of that story … you feel a bit special”* (FG1). The experiences of being seen, heard, and confirmed were important for feeling part of a social network, and this promoted self-confidence. In this context, participants encouraged teachers and parents to increase their knowledge and insight into how adolescents experienced social support and confirmation by being part of a social network on various digital platforms. This call was expressed through the following quote*:* “*Many parents haven’t really got into it, they don’t know what we are doing in our worlds on social media*” (FG5).

## Discussion

It is crucial to enhance our understanding of adolescent interactions and behaviours on social media platforms, as these digital environments have become integral to the social fabric of contemporary culture among adolescents. Importantly, social media use can also cause potential harm, and most published studies focus on the potential risks of social media use rather than its health-promoting aspects (Schønning et al., 2020). The aim of this study was therefore to explore the experiences of social media use among adolescents from a health-promoting perspective by using the theory of salutogenesis as a theoretical framework.

The findings indicate that the participants experienced social media as a necessary social arena where they can communicate with each other at any time and that conversations or what happens on social media continue to be “topics” in the offline world. The participants expressed that social media provided social support by promoting the experience of unity and inclusion and that social media gave them access to a larger social network and prevented loneliness; hence, the present study shows that social media can be viewed as a health-promoting arena. Further, our findings show that social media provided adolescents with attention, acceptance, and confirmation from others, which promoted their self-esteem. The participating adolescents experienced an interplay between social support and self-esteem since support from their friends and others on social media promoted their self-esteem and made them feel better. Further, their positive feelings encouraged them to express themselves more, which in turn provided more social support, creating a positive feedback loop.

### Identity and SOC development

In the present study, the participants expressed that adolescence can be experienced as challenging and sensitive time for their identity development. From a salutogenic health-promoting perspective, identity is a crucial GRR. Therefore, it is especially important to focus on identity to promote SOC during this important developmental period (Braun-Lewensohn et al., 2022). Further, in salutogenesis, social support is one of the basic and most important GRR for the development of a stronger identity and SOC (Langeland & Vinje, 2022). Close social relations can help relieve tension by transforming it into coping, strengthening identity and SOC (Antonovsky, 1987, Langeland & Vinje, 2022). Further, social relations can help individuals view the world as making sense cognitively, emotionally, and instrumentally. Therefore, individuals can see themselves as having a central role in positively moving along the health continuum, thus promoting identity and SOC (Antonovsky, 1996). Nearly all the participants in the present study expressed having experiences of social support by receiving and providing emotional support via social media. As various social relationships are crucial for identity development (Langeland & Vinje, 2022), access to social media platforms can be an important GRR that helps strengthen adolescents’ identity development. Support, attention, feedback, and good conversations with several others on social media made the participants feel good about themselves, strengthening their self-esteem. This is consistent with the results from the European Union (EU) Kids Online 2020, a survey of 19 countries in which most Norwegian children reported that they experienced the internet as a positive social environment in which they felt safe (Smahel et al., 2020). This finding is also compatible with findings from a previous study that concluded that communication via social media provides an experience of social and emotional support (O’Reilly et al., 2018), and with a survey among Norwegian adolescents that indicated that receiving social support is common after sharing difficult subjects on social media (Kysnes et al., 2022). Weiss (1974) shared Antonovsky’s view of social support and asserted that social support is a contributing factor to satisfying people’s needs in relation to others, which in turn gives people an experience of security and trust. Confirmation of self-worth is, according to Weiss (1974), one of six social needs that might be fulfilled when high-quality social support is experienced (Langeland & Vinje, 2022). In the present study, the participants expressed that receiving validation from others via social media promoted their self-esteem. This, in turn, led them to express themselves more openly and distinctly, which facilitated social support. According to Weiss (1974), social integration is another important need related to the experience of social support (Langeland & Vinje, 2022). Interestingly, in the present study, the participants used social media as a resource where they maintained contact with others, thus facilitating social integration. This might promote better identities, SOC, and mental health. This finding is in line with findings from another study among Norwegian children (aged 9–16 years) suggesting that there is a positive relationship between time spent online and self-reported life satisfaction (Milosevic et al., 2024). According to the participants in our study, social media made it easier to be part of a group or network and provided an experience of being in control and having an overview of what is going on among their peers. Experiences of unity and inclusion amounted to feelings of social support, a finding echoed in another study, which suggested that social media makes it easier to interact with others and gives people an experience of belonging (Castellacci & Tveito, 2018). Furthermore, interestingly, some of the participants described social media as a positive resource that prevented loneliness and negative thoughts, which they believed to be health promoting. This is in line with findings from other studies that claim that the use of social media among adolescents reduces experiences of loneliness and isolation (Booker et al., 2018), and negative thought patterns (von Tetzchner, 2012). Along with previous findings, the present study suggest that social media can be a health-promoting resistance resource where adolescents have other people readily available while also facilitating the opportunity to maintain relationships and influence their own identity – possibly promoting SOC. Thus, it is reasonable to believe that emotional and social support on social media can be a health-promoting experience with an impact on identity development and SOC.

According to salutogenesis, the strength of one’s SOC is promoted by one’s ability to use different GRRs and thus have experiences characterised by consistency, underload/overload balance, and participation in decision-making (Antonovsky, 1996). Intelligence, understanding, and knowledge are considered as cognitive resources in salutogenesis (Antonovsky, 1987). According to the participants, social media is a social arena where they can talk to others who have similar interests, have discussions, or become motivated. Through the use of actions on social media, adolescents promoted experiences of understanding, mastery, and meaning. Further, they noted that participation in communities via social media allowed for communication between several adolescents where they could discuss and gain increased understanding and knowledge. It is important for people to comprehend the situations they are in during identity development. This knowledge can provide insight, helping adolescents become more aware of their own choices by feeling heard. The choices people make influence their identity, an important GRR for developing SOC (Idan et al., 2022; Langeland et al., 2016). According to Antonovsky (1996), people can experience the life situation they are in as understandable if the context they are in is clear and structured. The adolescents experienced social media as a different, global, and more accessible mode of communication compared with traditional forms of interactions. Through social media, it became easier to maintain contact with friends, engage in conversations, and stay updated on each other’s lives. In this way, social media contributed to creating a clearer and more predictable social context, providing the adolescents with a sense of comprehensibility and oversight in their relationships. This illustrates Antonovsky’s argument that a structured and understandable context promotes a sense of coherence, mastery, and security in life situations. In turn, experiencing support from others could help people cope with the situation they are in and create motivation for action. Clarity can thus promote social competence, which can strengthen SOC (Langeland et al., 2016). In our study, the participants described social media as a positive resource during a developmental stage that they personally experienced as particularly challenging. The participants highlighted that many adolescents struggle with self-esteem during this period, and that support from friends through the use of social media could contribute positively to their self-esteem. Social media could thus help them understand each other by providing a platform for sharing thoughts, feelings, and experiences, which could lead to individuals’ feelings being understood, thereby strengthening their identity. This finding is consistent with a study that stated that social media promotes new forms of identity formation by reducing the significance of physical space and other traditional physical boundaries that have previously structured everyday life (Brandtzæg, 2019). By transcending geographical distance and fixed social settings, social media enables individuals to construct, express, and negotiate their identities across flexible and digitally mediated contexts. Social media differs from physical social arenas in that it is less constrained by time and space, and by providing users with a greater array of social participation and self-presentation. This may enable new forms of social interactions and identity development. By allowing socialisation independent of physical encounters, social media makes relationships more accessible and continuous, which may reduce social isolation and strengthen experiences of belonging and social support. For adolescents in particular, increased accessibility and self-determination in social interaction can contribute to greater coping, security, and psychological well-being. This is supported by the developmental psychologist, von Tetzchner (2012), who noted that physical encounters play a smaller role for adolescents now than they previously did, as social media has changed adolescents’ social patterns in the way they contact each other. The opportunity to create new friendships independent of physical encounters is consistent with our findings that the participants found it easier to make new friends through their access to a larger social network on social media, thus promoting identity and SOC. Accordingly, the present study indicates that identity is not only developed and formed outside of social media, but also affected by adolescents’ use of social media. Accordingly, the development of identity and SOC could be crucial GRRs irrespective of whether they are experienced online or offline.

### Creating meaning and promoting coping and SOC

The present study shows that participation on social media may promote meaning in life. Meaning may be seen as the truest expression of being human, and it is the most important dimension in the SOC because it is an emotional component that includes motivation, engagement, energy, and participation in shaping outcomes, which contribute to help individuals make the best of their situation (Antonovsky, 1987; Langeland & Vinje, 2022). Feelings, existential issues, activities, and social support are crucial areas for creating meaning (Antonovsky, 1987). The present study indicates that the participants had the opportunity to invest in these spheres on social media. By using the different functions offered by social media, the participants experienced commitment, finding solutions, entertainment, and knowledge. The adolescents provided each other with feedback, advice, guidance, and contact. Further, the study participants found inspiration from others on social media and described digital communication as a motivational resource when life felt difficult. They noted that social media facilitates social support by providing the opportunity to communicate with others regardless of time and place. Further, they expressed feelings of being heard and understood. A central finding from our study is that adolescents draw upon their activity competence to navigate and engage with the structured interactions afforded by social media, addressing a variety of needs and purposes. It is therefore reasonable to suggest that social media can serve as a resource for adolescents by contributing to the enhancement of their self-esteem, which in turn may encourage greater openness and clearer self-expression. This may further influence adolescents’ engagement in meaningful activities related to school, leisure time and personal interests. Such engagement may foster a sense of coherence, contribute to the experience of meaning, and enhance adolescents’ overall coping capacities.

When life feels difficult, or when extra motivation is required to act in demanding situations, it is important to have personal and collective support available. These are important GRRs that help individuals become motivated, feel mastery, find solutions, and thus gain experience of meaningfulness, which again might promote SOC. According to the salutogenic theory, the more personal and collective resistance resources available to individuals, the higher the degree of SOC (Langeland & Vinje, 2022). The study indicated that the participants valued the ability to maintain contact and unity with others, not only through physical meetings but also through digital communication platforms. Social media can therefore be understood as a potential GRR that supports the experience of meaning and coherence in life and further possibly a higher SOC.

Accordingly, the participants put forth the notion that parents, schools, and others should be told that social media activity does not necessarily cause poor mental health, but rather that it might be an important social and globalised arena for building good, meaningful social networks and for promoting self-esteem. This is in line with findings from other studies that highlight the important role played by digital technology in children’s lives. The use of social media might have significant positive effects, as it allows adolescents to express themselves and engage authentically while working on digital platforms (Milosevic et al., 2024) and thus find it easier to be open on social media (Booker et al., 2018).

### Methodological considerations

#### Strengths and limitations

This study is limited by its focus on social media use at a general level, without distinguishing between specific platforms. Different platforms may entail different interaction patterns and potential health effects. Lincoln and Guba (1985) elaborated on four criteria for promoting trustworthiness in qualitative research: credibility, transferability, dependability, and confirmability. To achieve credibility in the present study, adolescents who had experiences with social media were recruited. Open-ended questions were employed to encourage participants to share a wide range of perspectives and experiences, allowing for the collection of broad and exploratory data. Our analysis was described above, and the findings were supplemented by quotes. To facilitate transferability, detailed descriptions of the context, selection, data collection, and analysis were provided. Findings have been presented in a thorough and substantiated manner.

Dependability is a prerequisite for credibility and refers to the stability of data, meaning that another researcher would achieve the same findings with the same participants (Polit & Beck, 2017). The interviewers were conscious about their own preconceptions by posing open questions, listening carefully, following up on topics that appeared, and summing up to ensure that they understood correctly. To achieve trustworthy data, the first, second, and last authors read the interviews and took part in the data analysis.

To achieve valid findings in the present study, the authors reflected upon their preconceptions. It was therefore important for the researchers to reset themselves, set aside their own preconceived opinions and thoughts, and be open and curious about other reflections. This provided the researchers with new insights, understanding, and increased knowledge. In addition, the selection of informants, data collection, and analysis were described with the intention of providing sufficient and adequate information for the evaluation of the study’s methodological rigour.

Although some of the interview questions might seem slightly controlling, this was an intentional choice. Since earlier studies have mainly focused on the negative factors of social media, facilitating participants’ reflections on both the potentially positive and negative sides of social media is important. Although the interviews were conducted in 2019, they may still provide valuable insights into fundamental aspects of adolescents’ use of social media, such as communication between friends, identity development, entertainment, and social belonging. However, more recent data may have captured other patterns in platform use, beliefs and experiences. Further, the present study provides knowledge about salutogenesis in a new setting. Accordingly, the focus has been on processes that might be health promoting. Another strength is that the interview guide was developed in collaboration with an interdisciplinary research group, adolescents, and masters-level psychology scientist-practitioner students.

The study limitations are that there were relatively few participants and that they were recruited from a limited geographical area. Further, only adolescents who were interested in participation were recruited, which may have led to bias. The adolescents who wanted to participate may have been very active social media users, have strong feelings or opinions for or against social media, or be characterised by a higher openness to social interaction. Another limitation of the study is that coding was conducted by a single author, which may introduce bias. However, preliminary interpretations were discussed with co-authors familiar with the transcripts, helping to enhance the credibility of the findings. The present study’s findings are based on participants’ self-reports from focus group interviews and therefore reflect subjective perceptions. Consequently, their answers cannot be interpreted as direct evidence of the actual effects of social media use, which could be more accurately assessed using implicit measures.

## Conclusions

The main goal in salutogenesis is to determine what explains movement toward the healthy end of the health-disease continuum. The present study suggests that some aspects of social media use might serve as important GRRs by promoting adolescents’ social support and self-esteem, moving them towards the health end of the continuum and influencing the development of a more constructive identity and meaning. Thus, social media might contribute to making sense out of life and accordingly be an important GRR in a health-promoting, meaningful learning process, in parallel to activities outside of social media. Teaching adolescents to become aware of the opportunities that social media may offer and helping them gain “digital social competence” may contribute to increased health-promoting use of social media. Accordingly, social media is an important part of life for adolescents. It provides a different context and a new way for adolescents to relate to the world and should be considered an important social arena. Our findings indicate that the interaction between the attention and confirmation that adolescents receive from others and the social support adolescents experience on social media make them feel heard and accepted by those who they are. The participants described social media as a social arena with different norms, rules, and overall cultures than other offline social arenas. Social media makes it easier for individuals to express themselves and their opinions compared to social face-to-face interactions, where it challenging for them to express themselves as they wish. Our findings thus indicate that social media can be a positive resource and strengthen the development of identity and SOC when adolescents are being conscious of and can learn, make decisions, and use the available resistance resources offered by social media.

## Data Availability

To protect participant confidentiality, the focus group interview transcripts are not publicly available. However, further information regarding the transcripts may be obtained from the corresponding author upon reasonable request.
